# Superconductivity and improved electrical conduction in anti-ThCr_2_Si_2_-type *RE*_2_O_2_Sb and *RE*_2_O_2_Bi with pnictogen square net

**DOI:** 10.1016/j.isci.2022.104742

**Published:** 2022-07-09

**Authors:** Hideyuki Kawasoko, Tomoteru Fukumura

**Affiliations:** 1 Department of Chemistry, Graduate School of Science, Tohoku University, Sendai 980-8578, Japan; 2 PRESTO, Japan Science Technology Agency, Saitama 332-0012, Japan; 3 Advanced Institute for Materials Research and Core Research Cluster (WPI-AIMR), Tohoku University, Sendai 980-8577, Japan

**Keywords:** Condensed matter physics, Superconductivity, Nanomaterials

## Abstract

Layered compounds have shown rich physical properties such as high-temperature superconductivity. Recently, monatomic honeycomb lattice systems, such as graphene, have been studied extensively, whereas monatomic pnictogen square nets in layered compounds also exhibit interesting electronic properties owing to their unusual negative valence states. Among them, anti-ThCr_2_Si_2_-type *RE*_2_O_2_*Pn* (*RE* = rare earth, *Pn* = Sb, Bi) with monoatomic *Pn* square nets were recently found to exhibit interesting electronic properties such as superconductivity and high carrier mobility. In this article, we review recent studies on crystal structures, electronic properties, and thin-film growth of *RE*_2_O_2_*Pn*.

## Graphical abstract

**Figure:**
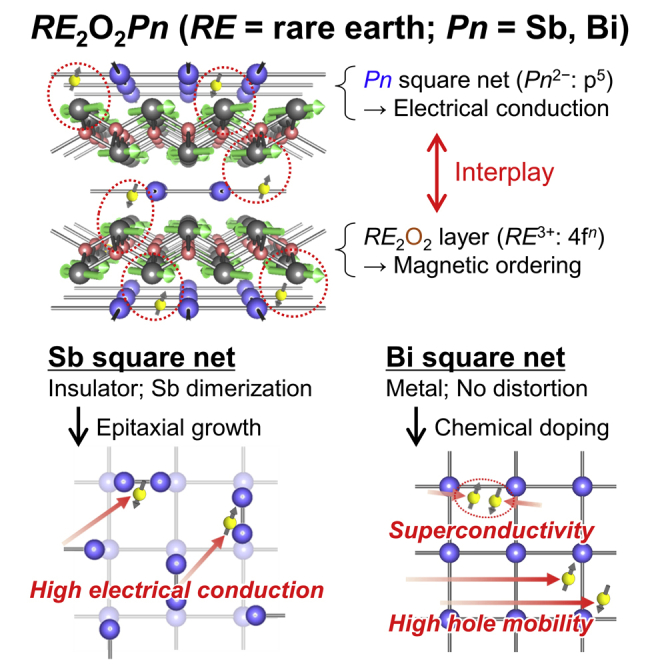

## Introduction

Layered compounds have been providing rich playgrounds for solid-state physics and chemistry because of their interesting physical properties such as high-temperature superconductivity in copper oxides through chemical substitutions (Klemm, 2012; Steglich et al., 1979; Armitage et al., 2010; Mackenzie and Maeno., 2003; Stewart, 2011; Qi and Zhang, 2011; Wilson et al., 1975; Wang et al., 2015; Ortiz et al., 2019; Ortiz et al., 2020). Indeed, La_2_CuO_4_, LaFeAsO, and FeSe became high-temperature superconductors by carrier doping via chemical substitution and intercalation (Bednorz and Muller, 1986; Kamihara et al., 2008; Guo et al., 2010). Also, chemical intercalation was used to make TiSe_2_ superconducting by suppressing the charge density wave (CDW) state and increasing the superconducting transition temperature in HfNCl by controlling the two-dimensional electronic states (Morosan et al., 2006; Kasahara et al., 2015).

Recently, monatomic layer compounds have attracted considerable attention owing to their exceptional electronic transport properties (Novoselov et al., 2004; Castro Neto et al., 2009). In particular, the honeycomb lattice system has been extensively studied for the exploration of new materials and properties (Mannix et al., 2017; Glavin et al., 2020). Also, monatomic Si, Sb, and Bi square nets are contained in layered ZrSiS, SrMnSb_2_, and SrMnBi_2_, respectively (Schoop et al., 2016; Liu et al., 2017; Benavides et al., 2018; Park et al., 2011). These square net compounds exhibit interesting electronic properties such as large magnetoresistance in ZrSiS and quantum Hall effect in EuMnBi_2_ and are expected to be topological materials owing to their unusual negative valence states (Ali et al., 2016; Masuda et al., 2016; Klemenz et al., 2020).

**Figure 1:**
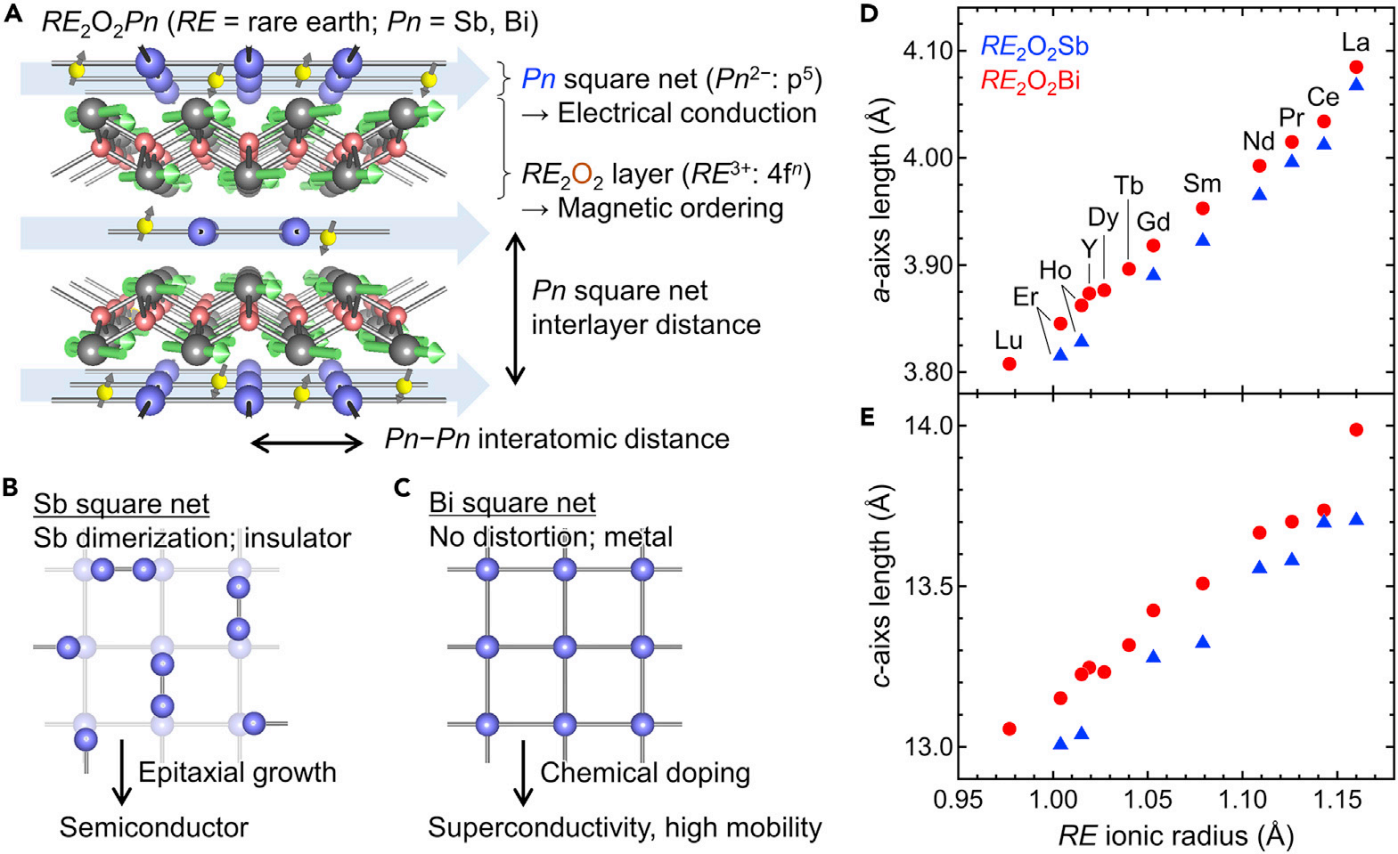
Crystal structure and lattice parameters of *RE*_2_O_2_*Pn* (A) Crystal structure of *RE*_2_O_2_*Pn*. The crystal structures are drawn by VESTA (Momma and Izumi, 2011). (B) Distorted Sb square net in *RE*_2_O_2_Sb. The crystal structures are drawn by VESTA (Momma and Izumi, 2011). (C) Bi square net in *RE*_2_O_2_Bi. The crystal structures are drawn by VESTA (Momma and Izumi, 2011). (D) *a*- and (E) *c*- axis lengths for *RE*_2_O_2_*Pn* as a function of the *RE* ionic radius (Shannon, 1976).

Anti-ThCr_2_Si_2_-type *RE*_2_O_2_*Pn* (*RE* = rare earth, *Pn* = Sb and Bi) are composed of monoatomic *Pn* square net and *RE*_2_O_2_ layer (Table 1 and Figures 1A, 1B, and 1C) (Benz, 1971). For both *RE*_2_O_2_Sb and *RE*_2_O_2_Bi, the *a*- and *c*- axis lengths increase with increasing *RE* ionic radius (Benz, 1971; Nuss and Jansen., 2009, 2012; Mizoguchi and Hosono, 2011; Wang et al. 2012; Sei et al., 2020), where the lattice constants of *RE*_2_O_2_Sb are smaller than those of *RE*_2_O_2_Bi for the same *RE* (Figures 1D and 1E). The electronic states of *RE*_2_O_2_*Pn* near the Fermi energy are composed of the *Pn* p-orbital with five valence electrons, where *Pn* is an unusual negative divalent ion (Kim et al., 2015, 2016). Interestingly, *RE*_2_O_2_Sb is an insulator, while *RE*_2_O_2_Bi is a metal despite the same electron configuration (Mizoguchi and Hosono, 2011; Wang et al., 2012; Wang et al., 2013). Recently, a superconducting transition was observed in *RE*_2_O_2_Bi by oxygen intercalation (Sei et al., 2016, 2020; Terakado et al., 2018). A dramatic decrease in electrical resistivity was reported in La_2_O_2_Sb by forming the epitaxial thin film (Yamamoto et al., 2021). These results imply a principal role of chemical approaches to manipulate the physical properties of *RE*_2_O_2_*Pn*. In this article, we review the fundamental properties and chemical modifications of *RE*_2_O_2_*Pn* bulk polycrystals in addition to the growth of *RE*_2_O_2_*Pn* epitaxial thin films. First, the fundamental properties, oxygen intercalation, chemical substitution, and thin-film growth of *RE*_2_O_2_Bi are described. Next, the fundamental properties and thin-film growth of *RE*_2_O_2_Sb are described.

**Table 1:** Summary of crystal structural parameters and physical properties for *RE*_2_O_2_*Pn*

		Y (4f^0^)	La (4f^0^)	Ce (4f^1^)	Pr (4f^2^)	Nd (4f^3^)	Pm	Sm(4f^4^)	Eu	Gd (4f^7^)	Tb (4f^8^)	Dy (4f^9^)	Ho (4f^10^)	Er (4f^11^)	Tm	Yb	Lu (4f^14^)
Sb (5p^5^)	*a* (Å)		4.067	4.012	4.017	3.965		3.992		3.890			3.828	3.815			
	*c* (Å)		13.71	13.70	13.71	13.56		13.32		13.28			13.04	13.01			
	Tetragonality *c*/*a*		3.369	3.414	3.413	3.419		3.397		3.413			3.409	3.409			
	Electric. Cond. (bulk)		Insulator	Insulator	Insulator	Insulator		Insulator		Insulator			Insulator	Insulator			
	Electric. Cond. (film)		Semicond.	–	–	–		–		–			–	–			
	Magnetism		–	–	–	–		–		–			–	–			
	Oxygen intercalation		–	–	–	–		–		–			–	–			
	Doping element		Sr	–	–	–		–		–			–	–			
Bi (6p^5^)	*a* (Å)	3.873	4.084	4.034	4.014	3.993		3.953		3.918	3.896	3.876	3.862	3.845			3.808
	*c* (Å)	13.25	13.99	13.74	13.70	13.67		13.51		13.42	13.32	13.23	13.23	13.15			13.06
	Tetragonality *c*/*a*	3.420	3.424	3.405	3.413	3.423		3.417		3.426	3.418	3.414	3.425	3.420			3.429
	Electric. Cond. (bulk)	Metal (SC)	Semicond.	Metal	Metal	Metal		Metal		Metal	Metal (SC)	Metal (SC)	Metal	Metal (SC)			Metal (SC)
	Electric. Cond. (film)	Metal	PM	AFM	AFM	–		–		–	–	–	–	–			–
	Magnetism	PM	–	Metal	–	–		–		AFM	AFM	AFM	–	AFM			–
	Oxygen intercalation	SS, CZO	SS	–	–	–		–		–	CaO	CaO	–	CaO			CaO
	Doping element	H, Li, F	Sr	–	–	–		–		–	–	–	–	–			–

Electric. Cond.: electrical conduction; Semicond.: semiconductor; SC: superconductor; PM: paramagnet; AFM: antiferromagnet; SS: solid-state oxidation; CZO: (Ce, Zr)O2 topotactic oxidation; CaO: CaO oxidation.

Compounds in gray cells have never been synthesized.

## Bi square net system: *RE*_2_O_2_Bi

### Fundamental properties

**Figure 2:**
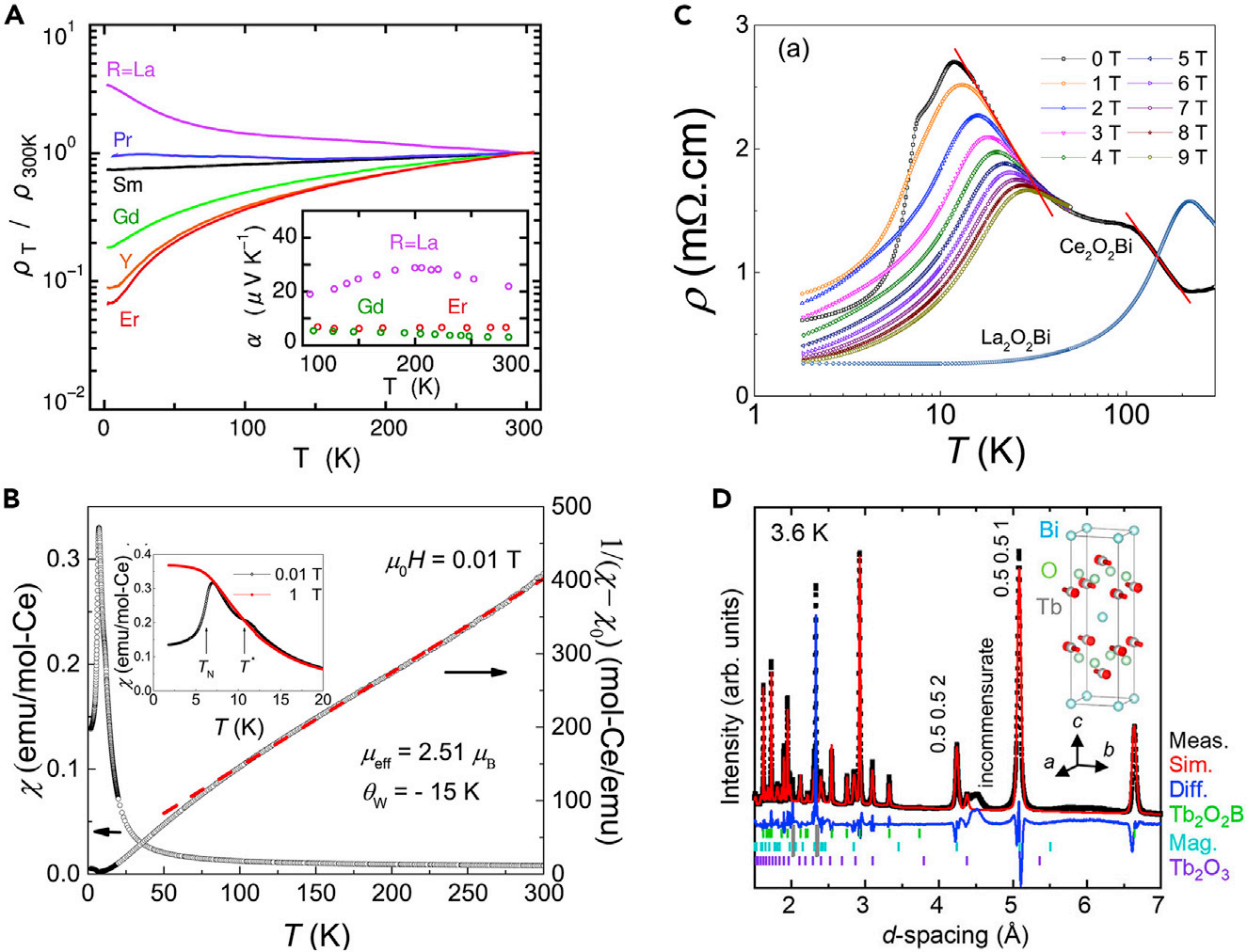
Physical properties of *RE*_2_O_2_Bi bulk polycrystals (A) Temperature dependence of the normalized electrical resistivity for *RE*_2_O_2_Bi bulk polycrystals (Reprinted with permission from Mizoguchi and Hosono, 2011 Copyright [2011] American Chemical Society). The inset shows the temperature dependence of the Seebeck coefficient for *RE*_2_O_2_Bi bulk polycrystals. (B and C) Temperature dependence of (B) magnetic susceptibility and (C) electrical resistivity for Ce_2_O_2_Bi bulk polycrystals (Reprinted with permission from Qiao et al., 2021a Copyright [2021] Elsevier). The inset of (B) shows the magnified figure. In (C), the electrical resistivity of La_2_O_2_Bi bulk polycrystal is also shown. (D) Neutron diffraction patterns of Tb_2_O_2_Bi bulk polycrystal and the Rietveld refinements for basis vectors of [1 −1 0] and [−1 1 0]. Light blue bars denote the magnetic reflection positions of the Tb_2_O_2_Bi phase. Gray areas with only Al peaks from the sample cells were excluded in the process of Rietveld analysis (Reprinted with permission from Kawasoko et al., 2019 Copyright [2019] AIP publishing).

In *RE*_2_O_2_Bi, the band near the Fermi energy is composed of the Bi 6p-orbital. The negative divalent Bi in the square net has one hole per Bi atom, resulting in p-type conduction, which is consistent with the positive Seebeck coefficient (inset of Figure 2A). The undistorted Bi square net in *RE*_2_O_2_Bi was beneficial for metallic conduction. However, the electrical resistivity increased monotonically with increasing *RE* ionic radius owing to the chemical pressure effect via an increase in the Bi-Bi distance of the square net, which resulted in semiconducting La_2_O_2_Bi (Figure 2A) (Mizoguchi and Hosono, 2011). From a theoretical study, the insulating behavior in La_2_O_2_Bi was attributed to strong electron-phonon interaction (Kim et al., 2016).

Typically, *RE*_2_O_2_Bi exhibits antiferromagnetic ordering owing to the 4f electrons in the *RE*_2_O_2_ layer. Consequently, conduction carriers in the Bi square net show unique magnetotransport behavior caused by the interaction between the conduction carriers and localized magnetic moments (Sei et al., 2020; Qiao et al., 2021a, 2021b). For example, Ce_2_O_2_Bi undergoes an antiferromagnetic transition at 6.2 K (Figure 2B). Additional anomalies in the magnetization and specific heat at 10.7 K suggested Kondo coherence behavior (Qiao et al., 2021a). The electrical resistivity of Ce_2_O_2_Bi increased from 190 K to 15 K owing to the Kondo scattering (Figure 2C). These behaviors were caused by the interaction between the conducting Bi 6p electrons and localized Ce 4f electrons. The carrier density of Ce_2_O_2_Bi was 7.9 × 10^20^ cm^−3^, which was smaller than that of the usual Kondo lattice compounds, suggesting that Ce_2_O_2_Bi could be a new platform for exploring Kondo physics.

Tb_2_O_2_Bi underwent an antiferromagnetic transition at 11.1 K and exhibited metamagnetic behavior below 5 K (Kawasoko et al., 2019). The magnetic structure of Tb_2_O_2_Bi was determined by neutron diffraction measurements (Figure 2D), indicating that the antiferromagnetic ordering originated from the Tb 4f electrons with a propagation vector of [0.5 0.5 0] (inset of Figure 2D). The observed incommensurate magnetic ordering was suggested to be the origin of the metamagnetic behavior of Tb_2_O_2_Bi (Kawasoko et al., 2019). From the electrical measurements, three anomalies were observed at 11, 28, and 35 K (Sei et al., 2020). The anomaly at 11 K was attributed to the suppression of magnetic scattering as the result of the antiferromagnetic ordering. Because there was no structural phase transition below 40 K, the anomalies at 28 and 35 K could be attributed to the crystal-field effect and Kondo scattering, respectively, as has been discussed for heavy fermion systems.

### Oxygen intercalation

**Figure 3:**
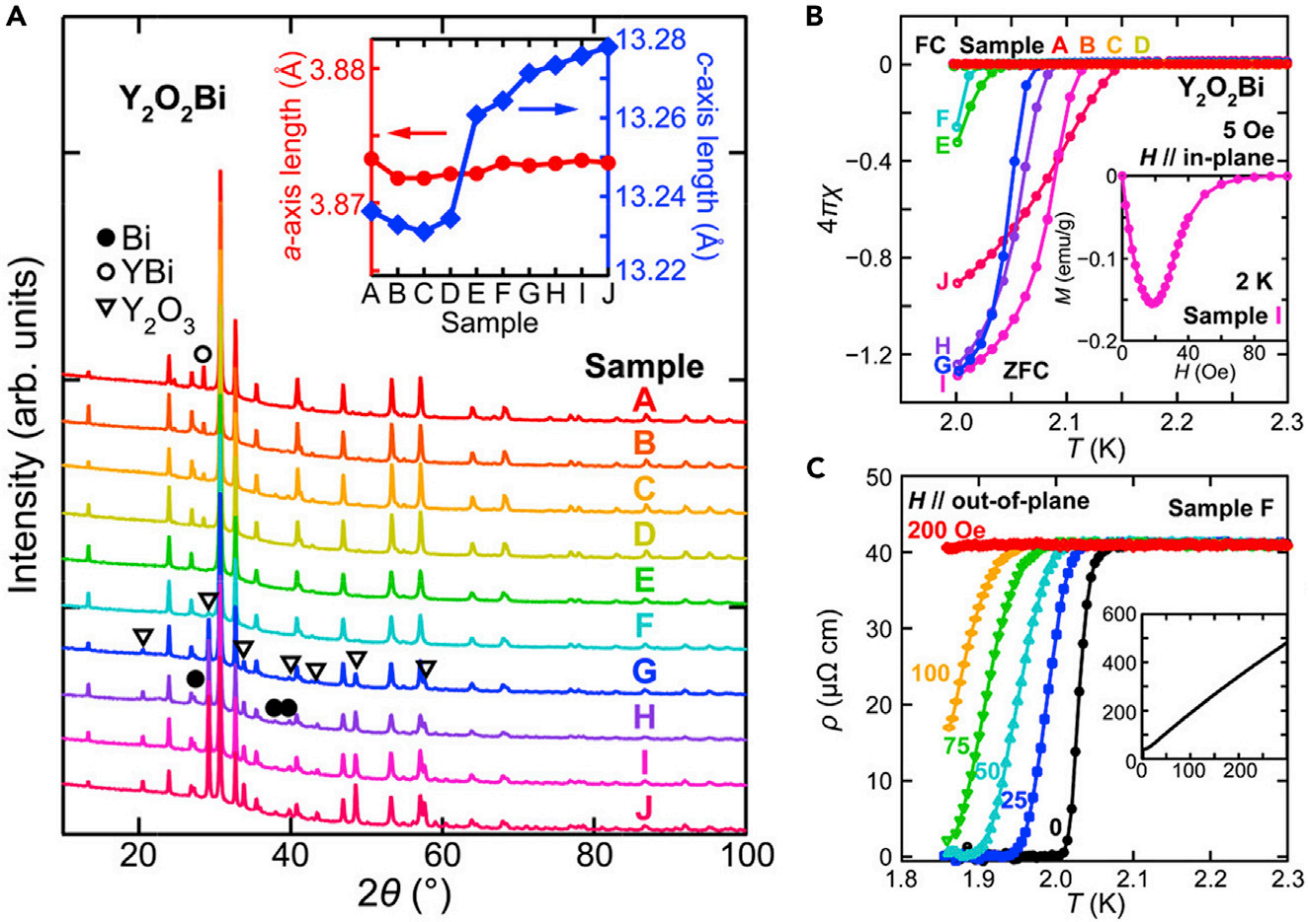
Oxygen intercalation and superconductivity in Y_2_O_2_Bi bulk polycrystals (A) Powder XRD patterns of Y_2_O_2_Bi bulk polycrystals. Samples A−J correspond to each nominal composition of Y_2_O_*x*_Bi_1.5_ (*x* = 1.1–2.0, each sample different by 0.1). Inset shows the lattice constant of each sample. (B) Temperature dependence of the magnetic susceptibility with zero-field cooling (ZFC) and field cooling (FC) processes at 5 Oe for samples A−J. The inset shows magnetization curve at 2 K for sample I. (C) Temperature dependence of the electrical resistivity near *T*_c_ and in the range of 1.85–300 K (inset) for sample F (Reprinted with permission from Sei et al., 2016 Copyright [2016] American Chemical Society).

In Y_2_O_2_Bi, the *a*-axis length was nearly constant at around 3.873 Å, whereas the *c*-axis length increased from 13.23 Å to 13.28 Å, with increasing the nominal amount of oxygen in the solid-state reaction (Figure 3A) (Sei et al., 2016). This uniaxial elongation along the *c*-axis indicated oxygen intercalation between the Bi square net and the Y_2_O_2_ layer in Y_2_O_2_Bi. Superconductivity emerged in the Y_2_O_2_Bi with *c*-axis length longer than 13.26 Å (samples E–J in Figures 3B and 3C). Because the change in the carrier density between the superconducting and non-superconducting Y_2_O_2_Bi was negligibly small, the superconductivity was unlikely caused by carrier doping. The superconducting transition temperature was correlated with the interlayer distance between the Bi square nets, i.e., a half of the *c*-axis length, suggesting that the origin of superconductivity is the increase in the two-dimensionality of the conducting Bi square net.

**Figure 4:**
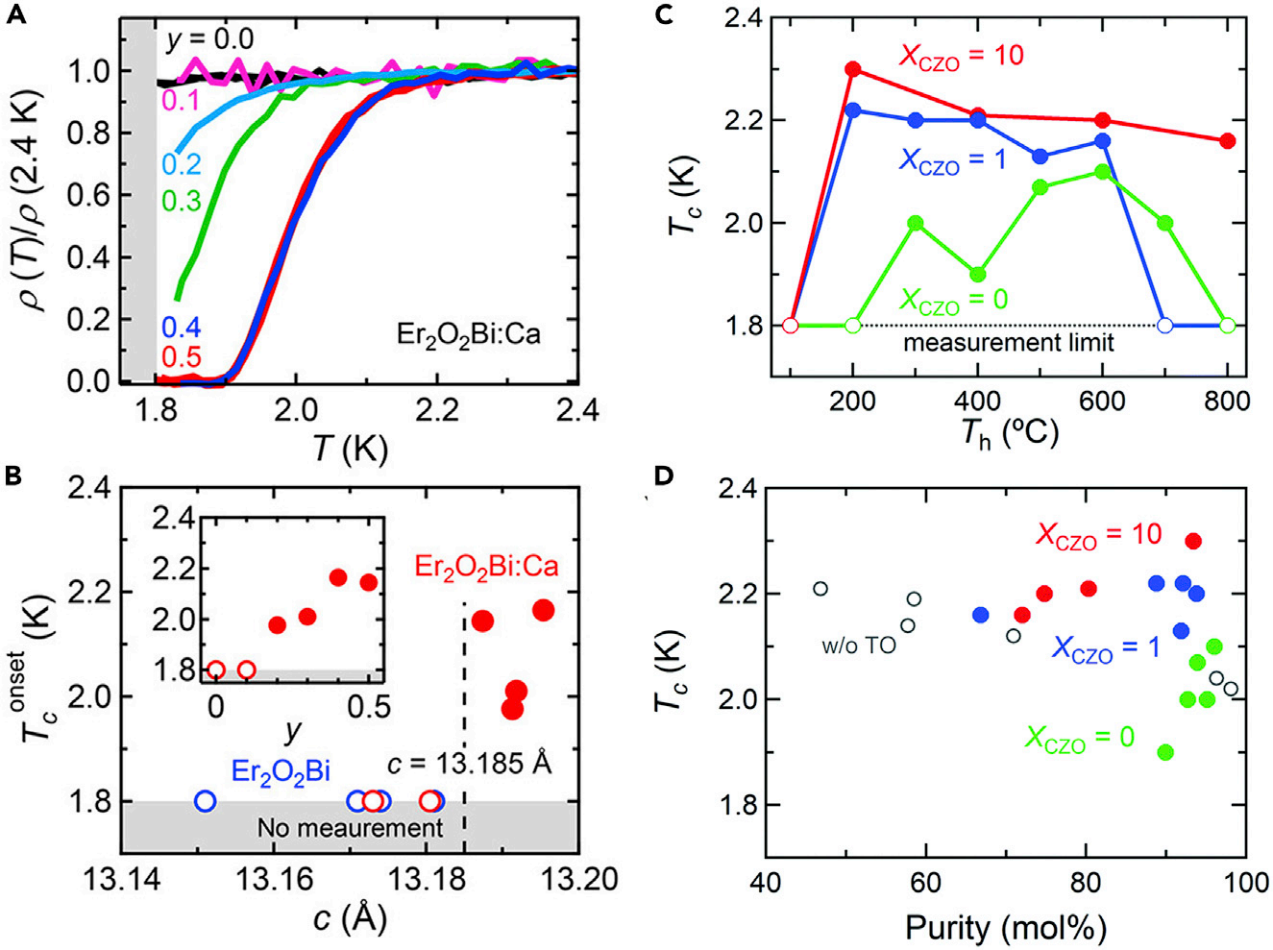
CaO and topotactic oxidations for superconductivity in *RE*_2_O_2_Bi bulk polycrystals (A) Temperature dependence of the normalized electrical resistivity for oxygen-intercalated Er_2_O_2_Bi polycrystals with various nominal compositions [*y*: Er_2_O_1.4_Bi_1.3_ + (CaO)_*y*_]. (B) Superconducting transition temperature (*T*_c_^onset^) as a function of the *c*-axis length for Er_2_O_2_Bi bulk polycrystals (Reprinted with permission from Terakado et al., 2018 Copyright [2018] American Chemical Society). The inset of (B) shows the *y* dependence of *T*_c_. Open circles denote no superconducting transition down to 1.8 K. (C) Heating temperature (*T*_h_) dependence of superconducting transition temperature (*T*_c_) for Y_2_O_2_Bi bulk polycrystals with various molar ratios of Zr-doped CeO_2_ (CZO) to Y_2_O_2_Bi (*X*_CZO_). (D) Relationship between *T*_c_ and the purity of Y_2_O_2_Bi bulk polycrystals after topotactic oxidation (TO) with various *X*_CZO_ (solid symbols) and after conventional solid-state oxidation (open symbols) (Reprinted with permission from Abe et al., 2021 Copyright [2021] the Royal Society of Chemistry).

In contrast to Y_2_O_2_Bi, oxygen was not intercalated in Er_2_O_2_Bi by increasing the nominal amount of oxygen in the solid-state reaction, resulting in no superconductivity (Terakado et al., 2018). However, CaO was found to serve as an oxidant for oxygen intercalation without chemical substitution in the solid-state reaction of Er_2_O_2_Bi; thus, the *c*-axis length of Er_2_O_2_Bi increased via oxygen intercalation. For the *c*-axis length longer than 13.185 Å, superconductivity emerged in Er_2_O_2_Bi (Figures 4A and 4B).

Recently, an oxidative topotactic method using the oxygen storage material Zr-doped CeO_2_ was developed, in which oxygen intercalation into Y_2_O_2_Bi was demonstrated (Abe et al., 2021). In contrast to oxygen intercalation via solid-state reaction at 1000°C, the oxidative topotactic method enabled oxygen intercalation into Y_2_O_2_Bi at a considerably lower temperature of 200°C, resulting in a superconducting transition (Figure 4C). This low-temperature process significantly improved the purity of the superconducting Y_2_O_2_Bi by over 90 mol%, and the superconducting transition temperature was slightly enhanced (Figure 4D).

**Figure 5:**
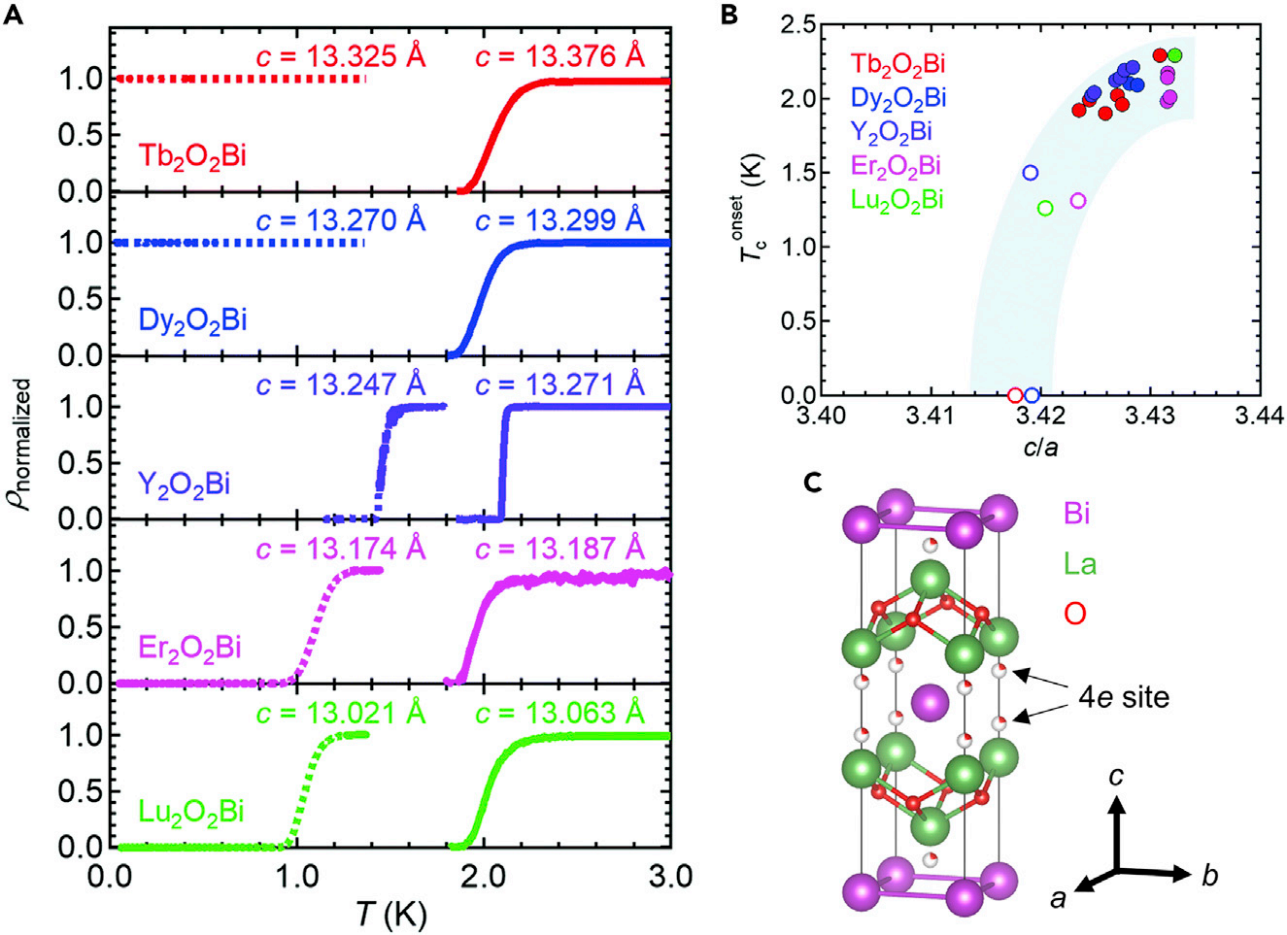
Superconductivity, tetragonality, and crystallographic site of intercalated oxygen in *RE*_2_O_2_Bi bulk polycrystals (A) Temperature dependence of the normalized resistivity for the stoichiometric (dashed curve) and oxygen-intercalated (solid curve) *RE*_2_O_2_Bi bulk polycrystals (*RE* = Tb, Dy, Y, Er, and Lu). (B) Relationship between the tetragonality *c*/*a* and superconducting transition temperature for *RE*_2_O_2_Bi bulk polycrystals (*RE* = Tb, Dy, Y, Er, and Lu). Open and solid circles correspond to the stoichiometric and oxygen-intercalated *RE*_2_O_2_Bi polycrystals, respectively (Reprinted with permission from Sei et al., 2020 Copyright [2021] the Royal Society of Chemistry). (C) Crystal structure of oxygen-intercalated La_2_O_2_Bi determined by Rietveld refinement on synchrotron powder XRD patterns (Reprinted with permission from Matsumoto et al., 2020 Copyright [2020] AIP publishing).

Electrical resistivity measurements at ultralow temperatures revealed Y_2_O_2_Bi, Er_2_O_2_Bi, and Lu_2_O_2_Bi to be superconducting even with stoichiometric oxygen (Figure 5A) (Sei et al., 2020). The other *RE*_2_O_2_Bi (*RE* = Tb, Dy) became superconducting by increasing the *c*-axis length via oxygen intercalation (Figure 5A). Interestingly, the tetragonality (*c*/*a*) of *RE*_2_O_2_Bi was found to govern the superconducting transition temperature irrespective of the presence or absence of magnetic ordering (Figure 5B), suggesting that the tetragonality is a key parameter for the superconductivity in *RE*_2_O_2_Bi. From synchrotron X-ray diffraction (XRD) measurements of oxygen-intercalated La_2_O_2_Bi, the crystallographic position of the intercalated oxygen was located at the 4e site adjacent to the *RE* position (Figure 5C) (Matsumoto et al., 2020). Magnetization measurements at ultralow temperatures of Er_2_O_2_Bi showed both a superconducting transition at 1.23 K and an antiferromagnetic transition at 3 K, indicating the coexistence of superconductivity and magnetism (Qiao et al., 2021b). Further studies on the effects of tetragonality and magnetism on the superconductivity in *RE*_2_O_2_Bi are expected in the future.

Theoretical study on Y_2_O_2_Bi suggested that the intercalated oxygen was energetically stable in the center of the Bi square net (Cheng et al., 2017). According to the theoretical study, the oxygen intercalation caused the presence of a flat band near the Fermi energy, which was attributed to the origin of the superconductivity in Y_2_O_2_Bi. However, this result was inconsistent with the experimental synchrotron XRD results, as described above (Matsumoto et al., 2020).

**Figure 6:**
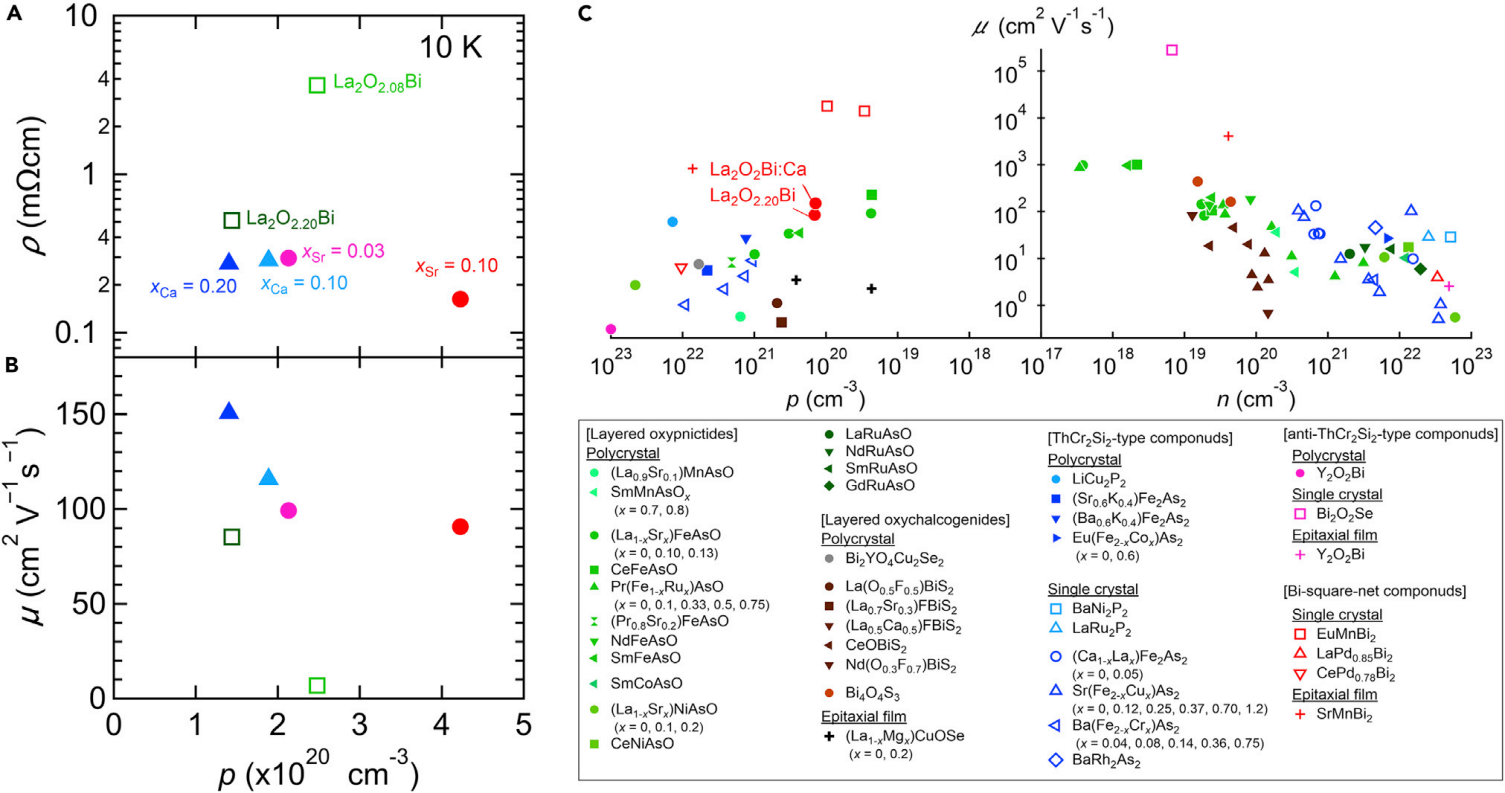
High hole mobility in La_2_O_2_Bi bulk polycrystals via oxygen intercalation and Sr substitution (A) Electrical resistivity and (B) hole mobility at 10 K as a function of the hole carrier density for La_2_O_2.08_Bi, La_2_O_2.20_Bi, La_2_O_2_Bi bulk polycrystals with *x*_Ca_ = 0.10 and 0.20, and La_2_O_2_Bi polycrystals with *x*_Sr_ = 0.03 and 0.10 (Reprinted with permission from Matsumoto et al., 2021 Copyright [2021] the Royal Society of Chemistry.). (C) Dependence of carrier mobility on hole (*p*) and electron (*n*) carrier densities for La_2_O_2_Bi bulk polycrystals and various layered oxypnictides and oxychalcogenides (Reprinted with permission from Matsumoto et al., 2020 Copyright [2020] AIP publishing). Solid, open, and cross symbols correspond to the data of bulk polycrystals, bulk single crystals, and epitaxial films, respectively. The plot of La_2_O_2_Bi:Ca is added to Figure 4 in Matsumoto et al., 2020.

Oxygen intercalation was performed not only in metallic *RE*_2_O_2_Bi but also in semiconducting La_2_O_2_Bi. Oxygen-intercalated La_2_O_2_Bi was prepared by controlling the nominal amount of oxygen in the solid-state reaction (Matsumoto et al., 2020). The oxygen intercalation did not induce superconductivity in semiconducting La_2_O_2_Bi, in contrast to other metallic *RE*_2_O_2_Bi (Matsumoto et al., 2021). However, the oxygen intercalation realized metallic La_2_O_2_Bi, originating from the improved hole carrier mobility, which was as high as 83 cm^2^/Vs. Oxygen intercalation was also performed using CaO as an oxidant, further increasing the *c*-axis length (Matsumoto et al., 2021). As a result, the mobility was enhanced up to 150 cm^2^/Vs (Figures 6A and 6B), which was nearly the highest value among other polycrystalline layered oxypnictides and oxychalcogenides (Figure 6C). Because the change in the carrier density of La_2_O_2_Bi via the oxygen intercalation was negligible, the high hole carrier mobility was possibly caused by the enhanced two-dimensionality of the Bi square net.

### Other chemical doping

**Figure 7:**
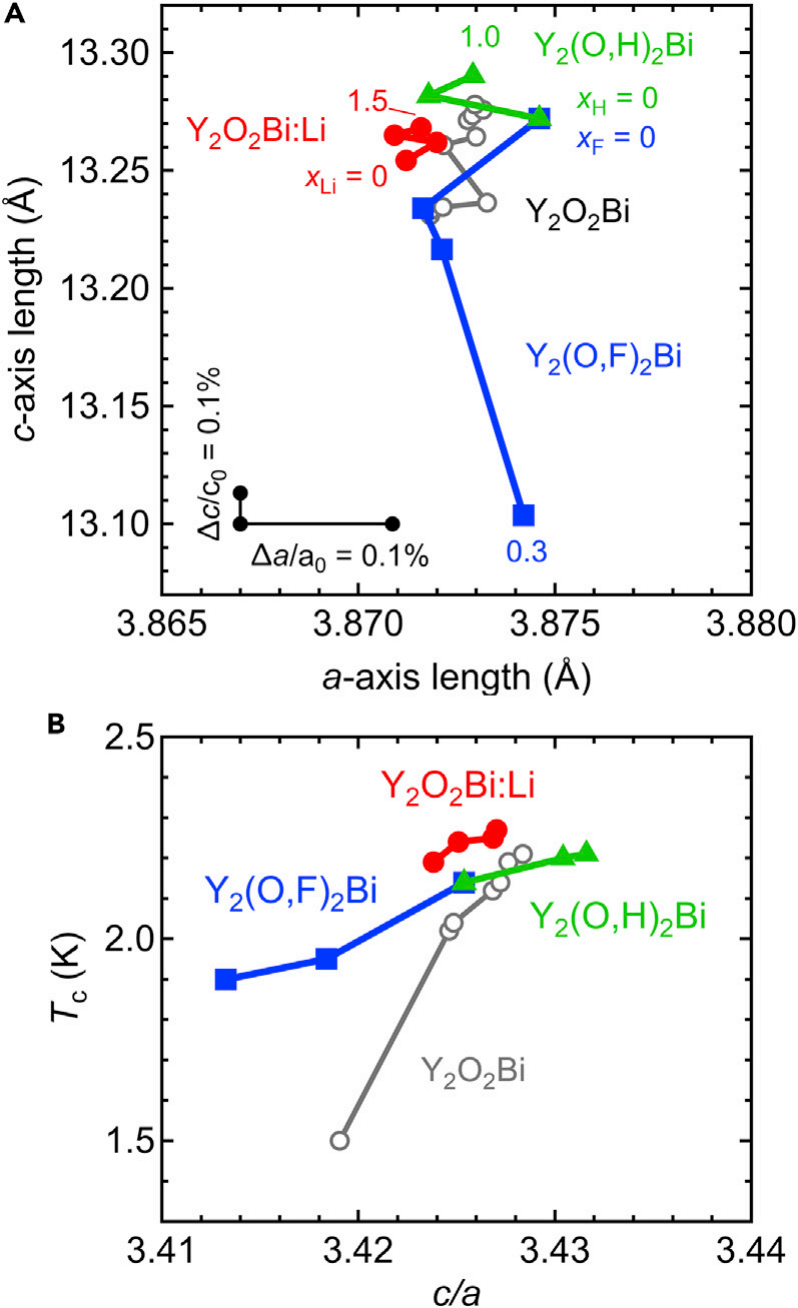
H substitution, Li intercalation, and F substitution in superconducting Y_2_O_2_Bi bulk polycrystals (A) *a*- and *c*-axis lengths for Y_2_(O,H)_2_Bi, Y_2_(O,F)_2_Bi, and Y_2_O_2_Bi:Li bulk polycrystals with different dopant contents. The minimum and maximum nominal H, Li, and F contents (*x*_H_, *x*_Li_, and *x*_F_, respectively) are displayed. Scale bars denote relative variations of *a*- and *c*- axis lengths (*a*_0_ = 3.867 Å, *c*_0_ = 13.10 Å). (B) Superconducting transition temperature (*T*_c_) as a function of *c*/*a* for bulk Y_2_(O,H)_2_Bi, Y_2_(O,F)_2_Bi, and Y_2_O_2_Bi:Li polycrystals with different dopant contents (Reprinted with permission from Terakado et al., 2022 Copyright [2022] the Royal Society of Chemistry). Gray plots denote the *T*_c_ of Y_2_O_2_Bi polycrystals with different oxygen contents.

Chemical doping other than oxygen intercalation was also effective in controlling the electrical properties of Y_2_O_2_Bi and La_2_O_2_Bi (Matsumoto et al., 2021; Terakado et al., 2022). For Y_2_O_2_Bi, H substitution for O, F substitution for O, and Li intercalation were reported. The H substitution and the Li intercalation increased the *c*-axis length, whereas the F substitution decreased the *c*-axis length. As a result, the variable range of *c*-axis length was increased in comparison with oxygen intercalation (Figure 7A). The superconducting transition temperature increased for the H substitution and the Li insertion but decreased for the F substitution. Furthermore, the superconducting transition temperature exhibited a monotonic increase as a function of *c*/*a*, as in the case of oxygen intercalation (Figure 7B). The superconducting transition temperature of the F-substituted Y_2_O_2_Bi was higher than that of the oxygen-intercalated Y_2_O_2_Bi even at the same *c*/*a*, suggesting an effect of electron carrier doping via F substitution on the superconducting transition temperature.

For La_2_O_2_Bi, La was substituted with Sr when La_2_O_2_Bi was co-sintered with SrO. Unlike in the case of co-sintering with CaO, not only oxygen intercalation but also Sr substitution occurred. As a result, the hole carrier density of La_2_O_2_Bi increased, in addition to the improved mobility via oxygen intercalation. Accordingly, the electrical resistivity of La_2_O_2_Bi was smaller than that of the La_2_O_2_Bi co-sintered with CaO (Figures 6A and 6B).

### Thin-film growth

In addition to *RE*_2_O_2_Bi bulk polycrystals, thin-film epitaxy of *RE*_2_O_2_Bi was investigated. Difficulties in the thin-film growth include realizing the unusual negative valence state of *Pn* and suppressing the re-evaporation of the highly volatile *Pn*. Indeed, the direct deposition method resulted in the thin-film growth of Bi-doped Y_2_O_3_. To overcome these difficulties, reactive solid-phase epitaxy has been developed (Sei et al., 2014).

**Figure 8:**
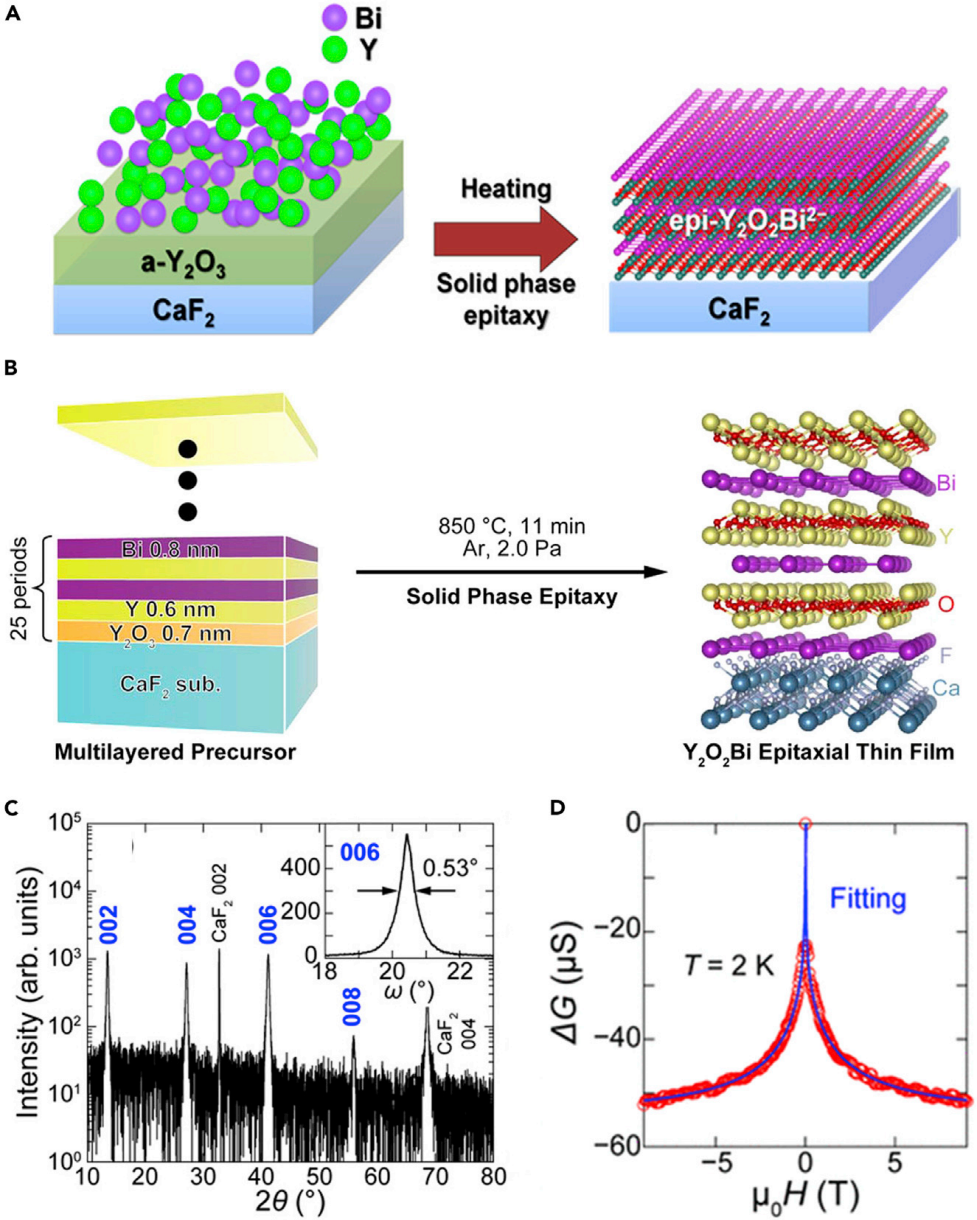
Growth methods, crystal structural analysis, and physical properties of Y_2_O_2_Bi epitaxial thin films Schematic illustration of (A) the reductive solid-phase epitaxy (Reprinted with permission from Sei et al., 2014 Copyright [2014] American Chemistry Society) and (B) the multilayer solid-phase epitaxy. (C) XRD *θ*-2*θ* pattern of the Y_2_O_2_Bi epitaxial thin film on CaF_2_ (001) substrate. The inset shows the 006 rocking curve. (D) Magnetoconductance at 2 K superposed with fitting curve by the Hikami-Larkin-Nagaoka model (Reprinted with permission from Sei et al., 2015 Copyright [2015] American Chemistry Society).

In the first study, an epitaxial thin film of Y_2_O_2_Bi was synthesized using reactive solid-phase epitaxy. Y and Bi powders on the Y_2_O_3_ amorphous film were heated on CaF_2_ (001) substrate under vacuum (Figure 8A), exhibiting weak 00*n* orientation XRD peaks of the Y_2_O_2_Bi thin film (Sei et al., 2014). This result indicated that the Y_2_O_2_Bi epitaxial thin film was synthesized on CaF_2_ (001) substrate by reactive solid-phase epitaxy, despite its insufficient crystallinity. To improve the crystallinity of the epitaxial thin film, a multilayer solid-phase epitaxy method was developed. (Sei et al., 2015). In this method, a multilayered thin film was used as a precursor for solid-phase epitaxy. For Y_2_O_2_Bi, multilayered precursor of Y, Bi, and Y_2_O_3_ layers prepared by magnetron sputtering was transformed into Y_2_O_2_Bi epitaxial thin film via *in-situ* heating (Figure 8B). The improved XRD peak intensity and narrow rocking curve indicated the high crystallinity of the Y_2_O_2_Bi epitaxial thin film (Figure 8C). Atomic force microscopy measurements revealed that the Y_2_O_2_Bi epitaxial thin film had a homogeneously flat surface. The negative valence state of Bi was confirmed using X-ray photoemission spectroscopy (XPS). The Y_2_O_2_Bi epitaxial thin film showed metallic electrical conduction, exhibiting electrical resistivity one order lower than that of the Y_2_O_2_Bi bulk polycrystal. The magnetoresistance of the Y_2_O_2_Bi epitaxial thin film indicated weak antilocalization attributed to the strong spin-orbit interaction of Bi (Figure 8D).

**Figure 9:**
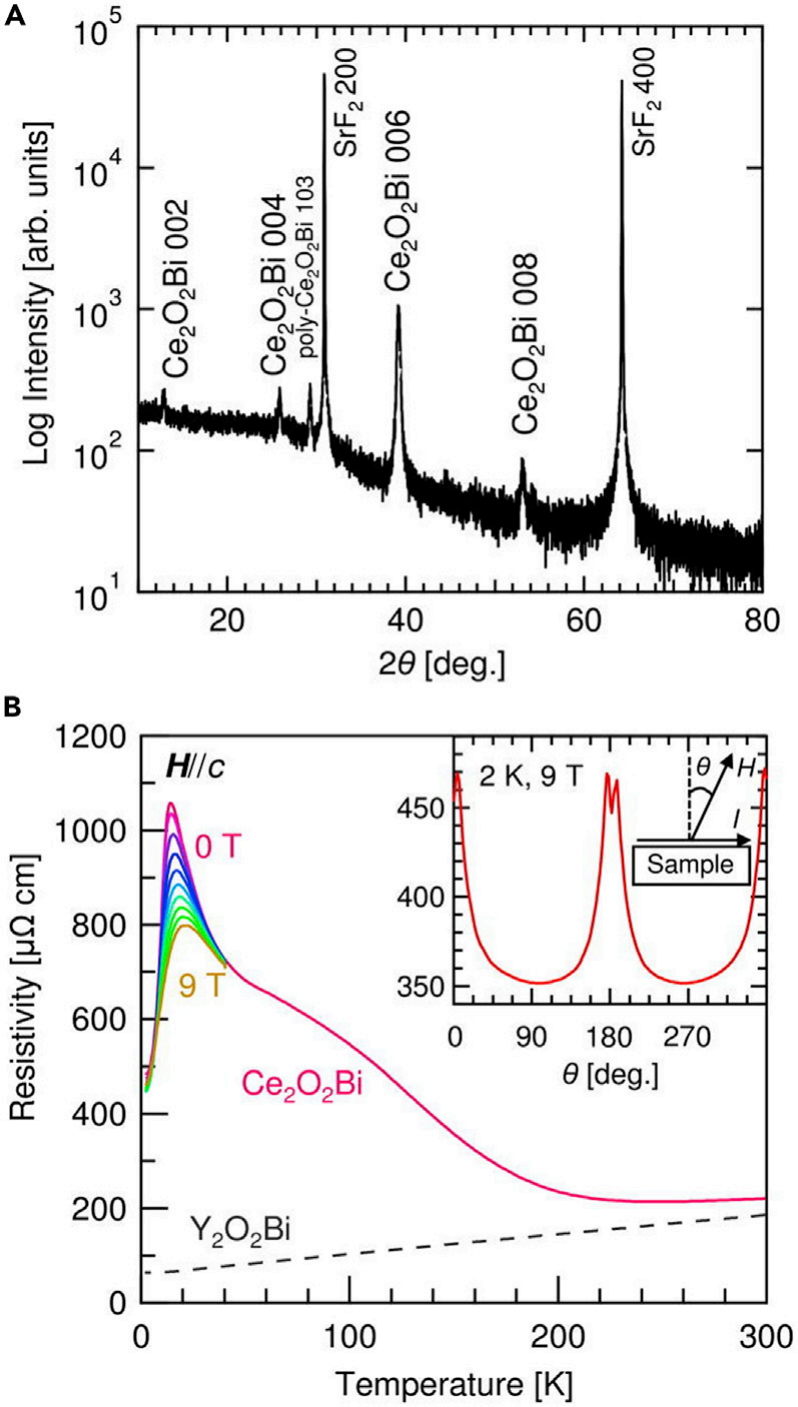
Crystal structural analysis and physical properties of Ce_2_O_2_Bi epitaxial thin films (A) XRD *θ*-2*θ* pattern for the Ce_2_O_2_Bi (001) epitaxial thin film on SrF_2_ (001) substrate. (B) Temperature dependence of electrical resistivity for the Ce_2_O_2_Bi and Y_2_O_2_Bi epitaxial thin films. The inset shows the angular dependence of the magnetoresistance at 2 K and 9 T for the Ce_2_O_2_Bi epitaxial thin films and the measurement geometry (Reprinted with permission from Shibata et al., 2017 Copyright [2017] AIP publishing).

Also, Ce_2_O_2_Bi epitaxial thin film was synthesized by the multilayer solid-phase epitaxy (Shibata et al., 2017). For Ce_2_O_2_Bi, the epitaxial thin film was synthesized on SrF_2_ (001) substrate using multilayered precursors of Ce, Bi, and CeO_2_ layers (Figure 9A). From XPS measurements, the valence state of Ce was found to be 3+ in Ce_2_O_2_Bi, confirming the presence of 4f electrons. The electrical resistivity at room temperature was approximately one-third of that of the Ce_2_O_2_Bi bulk polycrystal. In the Ce_2_O_2_Bi epitaxial thin film, the temperature dependence of the electrical resistivity showed a unique peak at 10 K that was suppressed under a magnetic field (Figure 9B) and was attributed to the interaction between the conducting Bi 6p electrons and the localized Ce 4f electrons, supported by a subsequent study of the Ce_2_O_2_Bi bulk polycrystals (Figure 2C) (Qiao et al., 2021a).

## Sb square net system: *RE*_2_O_2_Sb

### Fundamental properties

**Figure 10:**
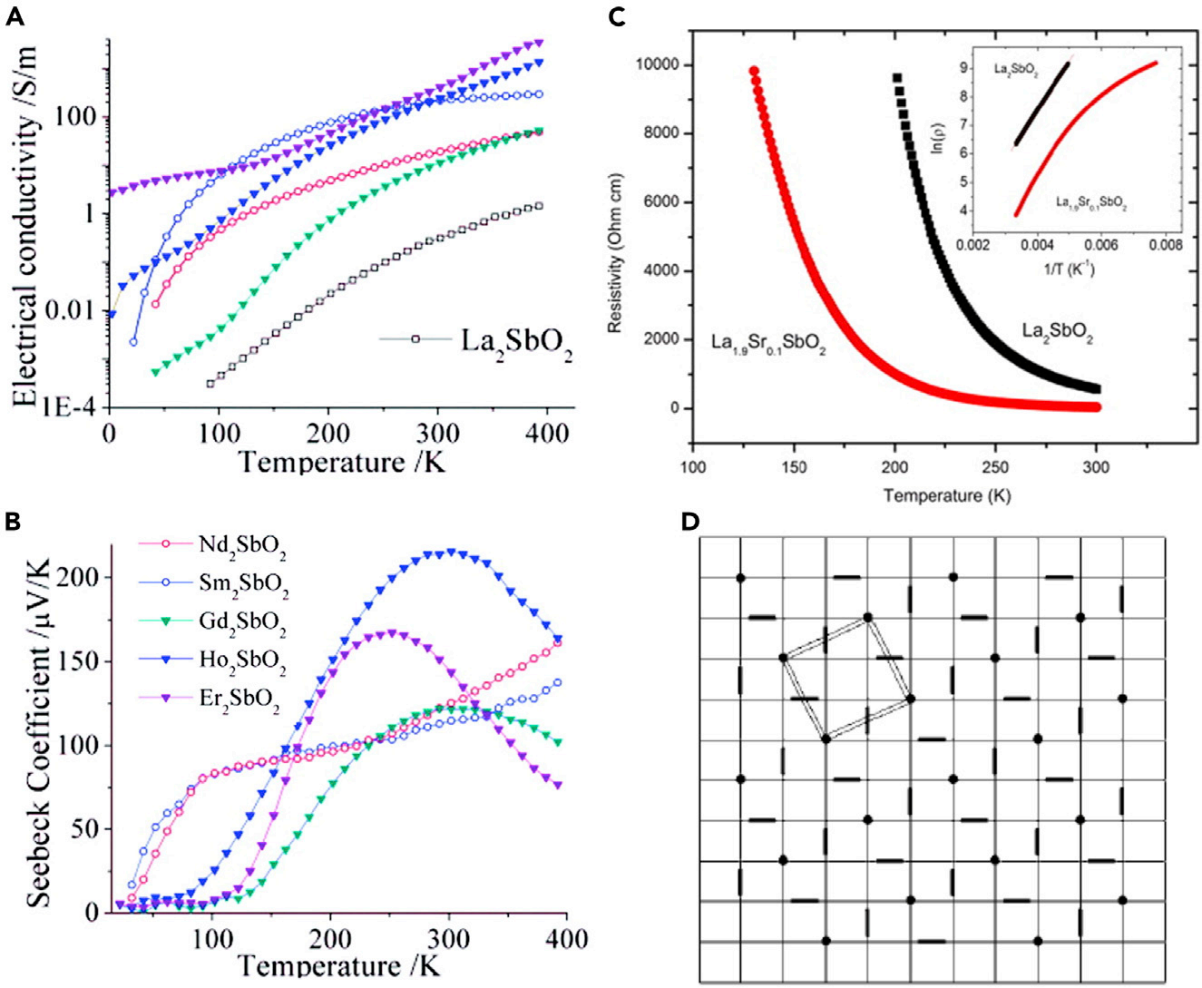
Physical properties and distorted Sb square net of *RE*_2_O_2_Sb bulk polycrystals Temperature dependence of (A) the electrical conductivity and (B) the Seebeck coefficient for *RE*_2_O_2_Sb bulk polycrystals (Reprinted with permission from Wang et al., 2012 Copyright [2012] American Chemistry Society). (C) Temperature dependence of the electrical resistivity for the La_2_O_2_Sb and La_1.9_Sr_0.1_O_2_Sb bulk polycrystals (Reprinted with permission from Muir et al., 2012 Copyright [2012] Elsevier). The inset shows the reciprocal temperature dependence of logarithmic electrical resistivity. (D) Schematic illustration of the distorted Sb square net (Reprinted with permission from Magdysyuk et al., 2013 Copyright [2013] International Union of Crystallography). Thick short lines and circle dots represent Sb dimers and isolated Sb atoms, respectively.

*RE*_2_O_2_Sb is an insulator despite of unclosed p^5^ electron configuration of Sb^2−^ in contrast to *RE*_2_O_2_Bi (Figure 10A). In *RE*_2_O_2_Sb except for La_2_O_2_Sb, the electrical conductivity increased owing to the decrease in Sb disorder, as *RE* ionic radius increased (Wang et al., 2012). Anderson localization was suggested as the origin of the insulating nature of *RE*_2_O_2_Sb because of the absence of a band gap from the band calculation of a superlattice structure (2*a* × *b* × *c*). Both the electrical conductivity and thermoelectric power increased with decreasing *RE* ionic radius, which was attributed to the electrical conduction mechanism depending on the *RE* ion (Figure 10B) (Wang et al., 2012). For La_2_O_2_Sb, (La_0.9_Sr_0.1_)_2_O_2_Sb was synthesized by solid-state reaction, and exhibited slightly lower electrical resistivity than stoichiometric La_2_O_2_Sb, indicating the improved electrical conduction possibly owing to hole doping (Figure 10C) (Muir et al., 2012).

The other crystal structural analysis and band calculations suggested that Sb dimerization in the square net generated the insulating conduction of *RE*_2_O_2_Sb (Nuss and Jansen, 2009; Kim et al., 2015; Magdysyuk et al., 2013). In *RE*_2_O_2_Sb, the atomic displacement parameter of Sb was strongly anisotropic in the *ab* plane, yielding two types of Sb-Sb distances. The detailed analysis of the XRD data of *RE*_2_O_2_Sb indicated the formation of a superlattice structure (4*a* × 4*b* × *c*), which was likely a result of Sb dimerization (Magdysyuk et al., 2013). The possible pattern of Sb dimerization was also suggested by the crystal structural analysis on Pr_2_O_2_Sb single crystal, as shown in Figure 10D.

**Figure 11:**
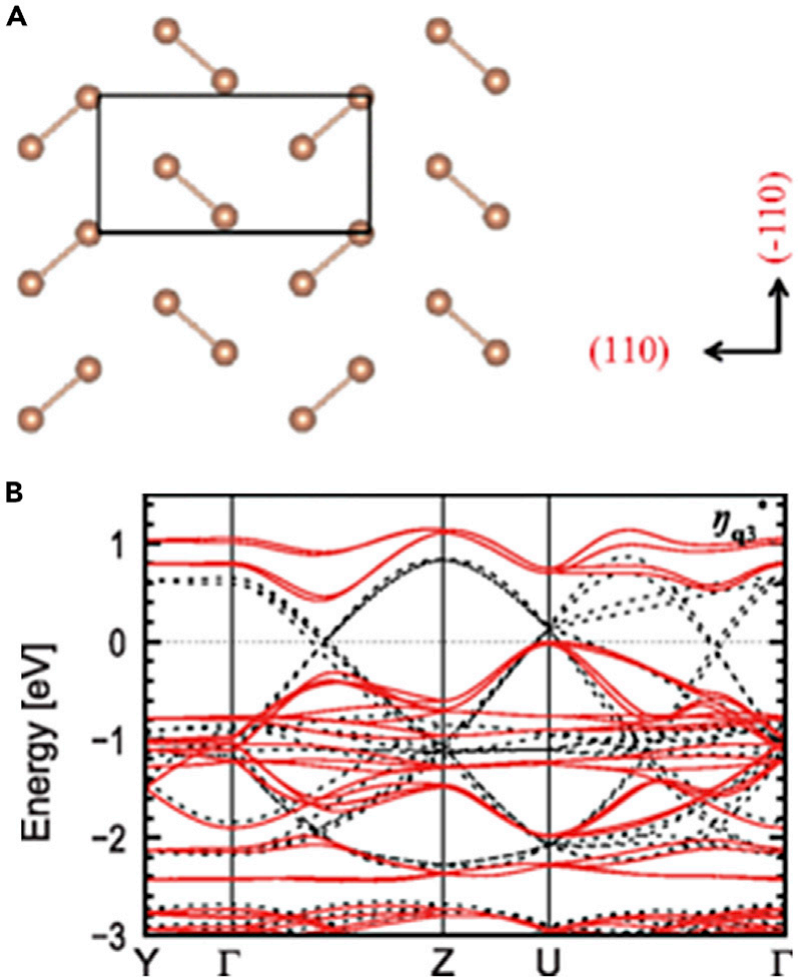
Sb Dimerization in *RE*_2_O_2_Sb (A) Schematic illustration of the herringbone-type Sb dimerization. (B) Band structures of La_2_O_2_Sb with (solid red) and without (dotted black) herringbone-type Sb dimerization (Reprinted with permission from Kim et al., 2015 Copyright [2015] American Physics Society).

To clarify the origin of the insulating nature of *RE*_2_O_2_Sb, the electronic structure and phonon dispersion were investigated by first-principles calculations (Kim et al., 2015). The nesting of the Fermi surface caused CDW instability, leading to the distortion in the Sb square net. From the band structures by assuming several types of structural distortions, a herringbone-type Sb dimerization was found to be the most stable (Figure 11A), where the band gap was estimated to be 0.42 eV (Figure 11B). In contrast, first-principles calculations suggested that Bi dimerization was unstable in La_2_O_2_Bi because the strong spin-orbit coupling owing to Bi modulated the Fermi surface, making the nesting less likely to occur (Kim et al., 2016). In the case of *RE*_2_O_2_Bi with smaller *RE* ionic radius such as Er_2_O_2_Bi, the nesting of the Fermi surface was suppressed by the volume contraction effect in addition to the spin-orbit coupling, preventing the distortion of the Bi square net.

### Thin-film growth

**Figure 12:**
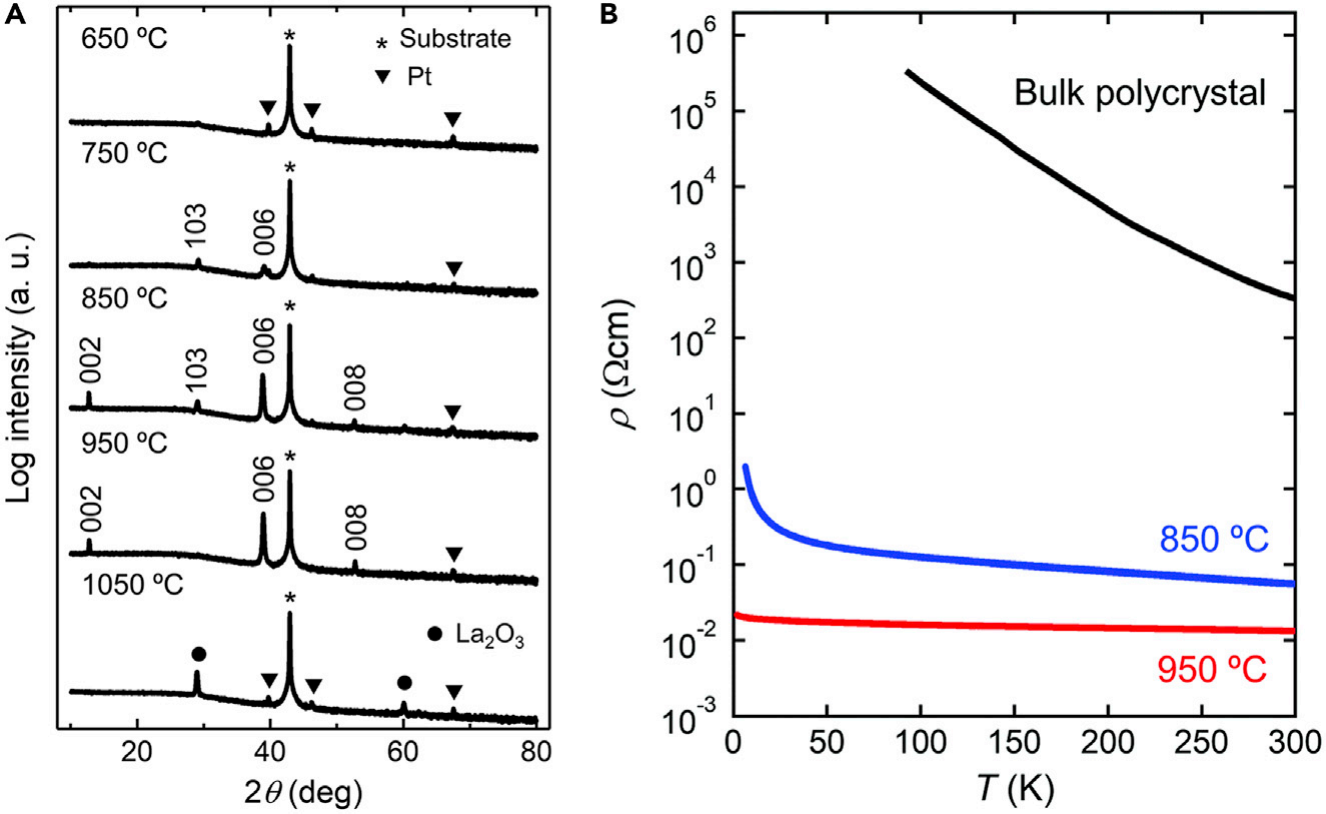
Crystal structural analysis and physical properties of La_2_O_2_Sb epitaxial thin films (A) XRD *θ*-2*θ* patterns of the La_2_O_2_Sb (001) epitaxial thin film on MgO (001) substrates for various growth temperatures. (B) Temperature dependence of the electrical resistivity for the La_2_O_2_Sb epitaxial thin films with *T*_g_ = 850 and 950°C (Reprinted with permission from Yamamoto et al., 2021 Copyright [2021] the Royal Society of Chemistry).

For *RE*_2_O_2_Sb, La_2_O_2_Sb epitaxial thin film was synthesized on MgO (001) substrate using multilayered precursors of La, Sb, and La_2_O_3_ layers (Yamamoto et al., 2021). From XRD results (Figure 12A), La_2_O_2_Sb was not crystallized by *in-situ* heating at 650°C, but became a highly crystalline epitaxial thin film with increasing the heating temperature to 850–950°C. The negative divalent state of Sb was confirmed by XPS. The Tauc plot of the absorption edge in the absorption spectrum indicated an indirect band gap of 0.17 eV, which was smaller than the band gap estimated by the band calculation with herringbone-type Sb dimerization (Figure 11B) (Kim et al., 2015). This result suggests that Sb dimerization in the film was suppressed. As shown in Figure 12B, the electrical resistivity was as low as 1/10000 of that of the bulk polycrystal (Wang et al., 2012). The significantly low electrical resistivity of the La_2_O_2_Sb epitaxial thin film was possibly caused by the suppression of Sb dimerization owing to epitaxial strain in addition to the reduction of the grain boundary scattering in the epitaxial thin film.

### Conclusion

In the anti-ThCr_2_Si_2_-type *RE*_2_O_2_*Pn* with monoatomic *Pn* square net, the electronic states of *RE*_2_O_2_*Pn* near the Fermi energy are composed of the *Pn* p-orbital with five valence electrons. *RE*_2_O_2_*Pn* are expected to be metals from the unclosed p^5^ electron configuration, which is seen in *RE*_2_O_2_Bi while *RE*_2_O_2_Sb is an insulator owing to Sb dimerization. By enhancing the tetragonality via chemical intercalation and substitution, *RE*_2_O_2_Bi exhibited superconductivity or high carrier mobility conduction. For the emergence of such unique electronic properties, the two-dimensionality of the Bi square net would be a key factor that is worth for theoretical investigation. On the other hand, the coexistence of superconductivity and magnetism in *RE*_2_O_2_Bi implies their unique magnetic phase diagrams, which are worth for both theoretical and experimental investigation. Also, the electrical resistivity of La_2_O_2_Sb was dramatically decreased by forming the epitaxial thin film, enabling the exploration of the electronic properties only in *RE*_2_O_2_Bi but also in *RE*_2_O_2_Sb. The thin-film epitaxy of *RE*_2_O_2_*Pn* could lead to further development of electronic and/or magnetic functionalities by tuning the lattice strain and dimensionality.
